# Protective effect of Naringenin on cisplatin-testicular damage through the oxidation and p38 MAPK inflammatory pathway

**DOI:** 10.5935/1518-0557.20250178

**Published:** 2026

**Authors:** Amirhesam Keshavarz Zarjani, Layasadat Khorsandi, Mohamad Heydari Kahkesh, Darioush Bijan Nejad, Farzaneh Mahmoudi Lafout, Mohammad Javad Khodayar, Atefeh Ashtari

**Affiliations:** 1 Cellular and Molecular Research Center, Medical Basic Sciences Research Institute, Ahvaz Jundishapur University of Medical Sciences, Ahvaz, Iran; 2 Department of Anatomical Sciences, Faculty of Medicine, Ahvaz Jundishapur University of Medical Sciences, Ahvaz, Iran; 3 Department of Toxicology, Faculty of Pharmacy, Ahvaz Jundishapur University of Medical Sciences, Ahvaz, Iran; 4 Toxicology Research Center, Medical Basic Sciences Research Institute, Ahvaz Jundishapur University of Medical Sciences, Ahvaz, Iran

**Keywords:** cisplatin, naringenin, oxidative stress, inflammation, MAPK, testis

## Abstract

**Objective:**

Cisplatin (CIS), a platinum-based chemotherapeutic, is highly effective in cancer treatment but often leads to severe adverse effects, including testicular damage and infertility. The oxidative stress and inflammation induced by CIS result in disrupted spermatogenesis, decreased testosterone levels, and testicular apoptosis, primarily mediated by elevated reactive oxygen species (ROS), pro-inflammatory cytokines, and activation of pathways like NF-κB and p38 MAPK. This study explores the protective role of naringenin (NG), a flavonoid with antioxidant, anti-inflammatory, and anti-apoptotic properties, in mitigating CIS-induced testicular damage in a murine model.

**Methods:**

Male mice were divided into control, CIS, NG, NG + CIS treated groups. Key outcomes included serum testosterone levels, oxidative stress markers, inflammatory mediators, Bax/Bcl-2 expression, histopathological evaluations, and morphometric analyses.

**Results:**

NG treatment attenuated CIS-induced oxidative stress by normalizing total oxidant status (TOS), total antioxidant capacity (TAC), and oxidative stress index (OSI) levels while reducing pro-inflammatory cytokines (TNF-α, IL-1β, IL-6) and NF-κB activation. NG also modulated apoptotic pathways by restoring the balance of Bax/Bcl-2 and suppressing p38 MAPK activation. Histological assessments revealed improved seminiferous tubule morphology and spermatogenesis in the NG+CIS group.

**Conclusions:**

NG effectively counteracts CIS-induced testicular toxicity, highlighting its therapeutic potential in preserving male fertility during chemotherapy by mitigating oxidative, inflammatory, and apoptotic damage.

## INTRODUCT

CIS is a well-known platinum-based chemotherapeutic agent introduced in the 1970s and is used to treat a variety of cancers (Fung *et al*., 2018; Romani, 2022). Despite its effectiveness in reducing tumor burden, numerous studies have indicated that it can cause cytotoxicity and severe damage to various tissues (Abd Rashid *et al*., 2021; Zhang *et al*., 2007). The clinical application of CIS has been limited due to the severe adverse effects it can cause, such as nephrotoxicity, neurotoxicity, and hepatotoxicity, particularly when administered in high doses and through repeated cycles (Kim *et al*., 2004; Sahar Khalil & Abeer, 2010; Shabani *et al*., 2012). Azoospermia, or permanent infertility, is another side effect observed in more than 50% of individuals treated with CIS (Abdel-Latif *et al*., 2022). In addition, a decrease in testicular weight, spermatozoa malformation, and steroidogenesis suppression have been documented (Ethics Committee of the American Society for Reproductive Medicine, 2005; Meistrich, 1999). CIS demonstrates cure rates as high as 80% for testicular cancer (Osanto *et al*., 1992). However, various reports indicate that CIS induces cytotoxicity in the germinal epithelium by lowering testosterone levels and elevating oxidative stress, which leads to the excessive release of reactive oxygen species (ROS) and lipid peroxidation (Fouad *et al*., 2017). The rise in the production of free radicals within cells is responsible for inducing oxidative damage in mitochondria and suppressing the activity of antioxidant enzymes (Coşkun *et al*., 2013). This ultimately triggers inflammation and apoptosis in testicular tissue (A A Aly & G Eid, 2020).

The overproduction of ROS initiates the oxidative stress cascade and reduces the levels of endogenous antioxidants. Consequently, an immune response is triggered, leading to inflammation. This inflammatory reaction induces cytotoxic effects in testicular tissue and overexpression of pro-inflammatory cytokines, such as tumor necrosis factor-alpha (TNF-α) (Taha *et al*., 2018). ROS activates nuclear factor-kappa B (NF-κB), which regulates inflammation and plays a vital role in oxidative and inflammatory tissue damage (Gao *et al*., 2021). CIS is recognized for initiating the MAPK pathway, which leads to the activation of the p38 enzyme (Arany *et al*., 2004). The p38 MAPK pathway contributed to inflammation, cell cycle regulation, and differentiation (Alhoshani *et al*., 2017; Koul *et al*., 2013). Activating p53 in response to CIS toxicity alters the pattern of transcriptional regulators, leading to DNA damage. It induces the transcription of the pro-apoptotic gene Bax, while simultaneously inhibiting the expression of the anti-apoptotic protein Bcl-2 (Han *et al*., 1999; Jiang *et al*., 2009).

Antioxidant mixtures are widely used as nutritional components and have been investigated for their capability to mitigate tissue and organ toxicities caused by various harmful substances. NG (4,5,7-trihydroxyflavanone), a flavonoid found in tomatoes, grapes, and citrus fruits, possesses biological and pharmacological properties (Chtourou *et al*., 2016). It plays a vital role in the detoxification of free radicals, and numerous studies have highlighted its anti-inflammatory, antioxidant, and anti-apoptotic effects (Felgines *et al*., 2000; Koyuncu *et al*., 2017; Martínez-Rodríguez *et al*., 2020). NG exhibits anti-inflammatory effects by regulating the expression of tumor necrosis factor-α (TNF-α) and transforming growth factor-β (TGF-β) (Amudha & Pari, 2011).

Considering the potential side effects associated with CIS and the resulting damage to testicular tissue, the current study explored the protective properties of NG against CIS-induced damage through antioxidant, anti-inflammatory, and anti-apoptotic pathways.

## MATERIALS AND METHODS

### Animals and Experimental Design

Thirty-two six-week-old male mice (25-30 g weight) were randomly divided into four separate groups, consisting of eight:

Group 1 (Control): Administer 0.1 mL of saline intraperitoneally for 14 days.

Group 2 (CIS): Intraperitoneal administration of saline (0.1 mL) and intraperitoneal injection of CIS (20 mg/kg) on the seventh day to induce testicular injury (Xiang *et al*., 2022).

Group 3 (NG): Intraperitoneal administration of NG (50 mg/kg) for 14 days (Fouad *et al*., 2019).

Group 4 (CIS + NG): Intraperitoneal administration of NG (50 mg/kg) for 14 days, followed by intraperitoneal administration of CIS (20 mg/kg) on the seventh day of the experiment.

Twenty-four hours after the final injection, the rats were anesthetized with an intraperitoneal injection of ketamine (50 mg/kg) and xylazine (5 mg/kg). Blood specimens were then collected and stored at -20°C. The testicles were harvested; the right testicle was preserved in formalin (10%) for histopathological examination, while the left testicle was stored at -80°C for measuring oxidative and inflammatory factors and western blot analysis (Mesbahzadeh *et al*., 2021; Othman *et al*., 2023).

### Testosterone Levels

Blood samples were collected under deep anesthesia. The serum was separated after clotting by centrifugation at 200 rpm for 15 minutes. Testosterone serum concentrations were measured using an ELISA testosterone kit (ARG80662, Taiwan).

### Inflammatory Factors

The testicular tissues were harvested, washed with PBS, and frozen at -80°C for ELISA analysis. Subsequently, the testicular tissues were homogenized in a cold buffer containing PBS (pH 7.4, 100 mM) and a protease inhibitor mixture. The homogenized specimens were centrifuged at 10,000 g for 20 minutes, and the supernatants were stored at -80°C until needed. The concentrations of NF-kB, TNF-α, IL-6, and IL-1β were determined according to the manufacturer’s guidelines of the ELISA kit (ZellBio, Germany).

### Oxidative Stress Markers

After homogenizing the testicular tissues, we measured the total oxidative status (TOS) and total antioxidant capacity (TAC) using Zellbio (Germany) kits. We then determined the ratio of TOS to TAC to calculate the oxidative stress index (OSI).

### RT-PCR

The levels of mRNA expression of Bax and Bcl-2 were assessed by extracting total RNA according to the manufacturer’s protocol (Super RNA Extraction Kit, Anacell, Iran). The RNA concentration was determined using a Nanodrop spectrophotometer. Real-time PCR was performed using the Real Q Plus 2x Master Mix Green (Ampliqon, Denmark) and the Step One Real-Time PCR Detection System (ABI, USA). GAPDH served as the reference housekeeping gene in the qRT-PCR analysis.

### Western Blotting

Testicular samples were homogenized in RIPA buffer containing protease inhibitors (Sigma, USA). After determining the protein concentration at 595nm (BioTek ELX800, USA), and separated by electrophoresis, proteins were relocated to a PVDF membrane, which was incubated with primary antibodies (p38 MAPK Antibody, Cell Signaling Technology, USA) for 24 hours and secondary antibodies for one hour. Protein detection was performed using a LI-COR Odyssey Infrared imaging system (LI-COR Bioscience, USA). ImageJ software was utilized to measure the grey values of the blots, and the relative protein levels were normalized to GAPDH.

### Histopathological Assessment

The right testicle was fixed. After preparation, the histological slides were stained with Hematoxylin and Eosin (H&E) to evaluate structural changes using a light microscope. The percentage of seminiferous tubules with vacuoles was calculated by dividing the number of affected tubules by the total number of healthy tubules in a field and multiplying by 100. Each testicle was assessed in at least 20 fields. The Johnsen’s score was utilized to assess the maturation of spermatocytes. In brief, the seminiferous tubules were ranked from 1 to 10, as detailed in the previous study (Johnsen, 1970). The diameter of the seminiferous tubules was measured using the Motic Images software program at a 400x magnification. This measurement was obtained by calculating the distance between the two basal membranes at opposite ends.

### Statistical Analyses

The data were analyzed using one-way ANOVA with GraphPad Prism 9 software and are presented as mean ± standard deviation. A *p*-value of less than 0.05 was considered statistically significant.

## RESULTS

### Testosterone

**Figure 1:**
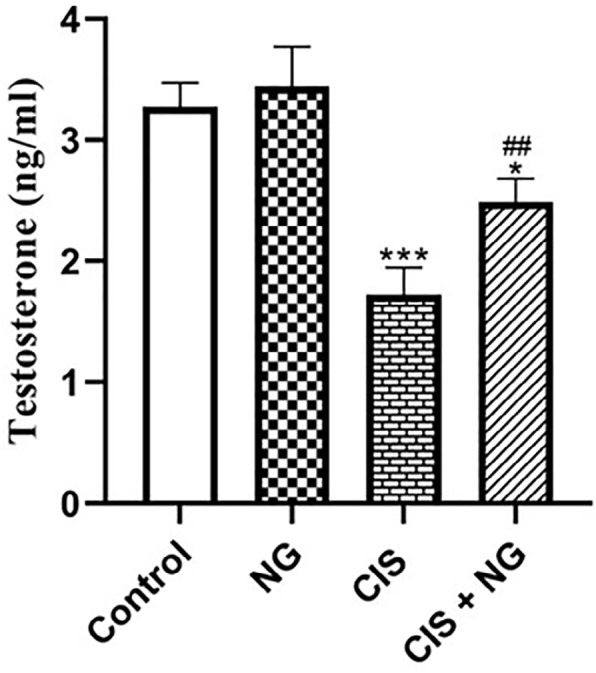
Testosterone levels in the different groups (Mean ± SD; n=8). * & ^#^ *p*<0.05, ** *p*<0.001. The asterisk and ^#^ symbols indicate a comparison to the control and CIS-intoxicated groups, respectively.

No significant difference was observed in serum testosterone levels of the NG and control groups. In the CIS-intoxicated animals, testosterone levels significantly declined compared to the control group (*p*<0.0001). However, in the NG + CIS group, testosterone levels were significantly higher than those in the CIS-intoxicated animals (*p*=0.0072) (Figure 1).

### Oxidative Stress Markers

The levels of TAC declined significantly in the CIS-intoxicated group and the CIS + NG group compared to the control group (*p*=0.0002 and *p*=0.0032, respectively). However, the TAC level in the CIS + NG group increased compared to the CIS-intoxicated group, although this increase was not statistically significant (*p*=0.153).

**Figure 2:**
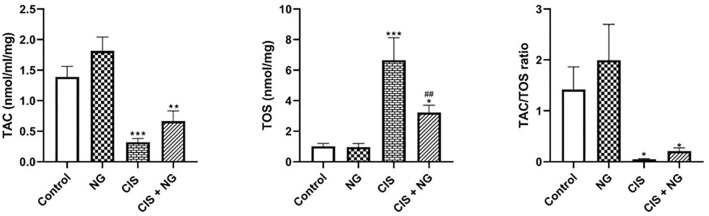
Oxidative stress biomarkers in different groups (mean ± SD; n=8). *& ^#^ *p*<0.05, ** & ## *p*<0.01, *** *p*<0.001. The asterisk and ^#^ symbols indicate a comparison to the control and CIS-intoxicated groups, respectivelyt.

The levels of TOS were significantly higher in both the CIS-intoxicated group and the CIS + NG group compared to the control group (*p*=0.0001 and *p*=0.0365, respectively). However, the level of TOS in the CIS + NG group was significantly lower than that in the CIS-intoxicated group (*p*=0.0032). The OSI increased in the CIS-intoxicated group (*p*=0.0011). However, the OSI significantly declined in the CIS+NG group compared to the CIS group (*p*=0.0047) (Figure 2).

### Inflammatory Parameters

IL-1β was significantly increased in both the CIS-intoxicated group and the CIS + NG group compared to the control group (*p*=0.0001 and *p*=0.0093, respectively). In the CIS + NG group, the concentration of IL-1β significantly decreased compared to the CIS-intoxicated group (*p*=0.0031). The concentration of IL-6 was increased in the CIS-intoxicated group compared to the control group (*p*=0.0002), and IL-6 significantly decreased in the CIS + NG group compared to the CIS-intoxicated group (*p*=0.0041).

**Figure 3:**
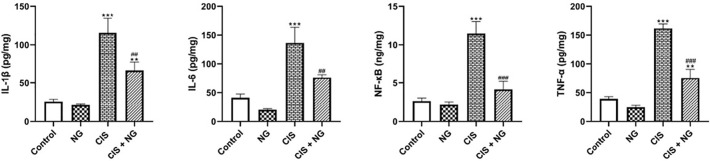
Effect of naringenin on testicular IL-1β, TNF- α, NF-κB, and IL-6 activity in CIS-treated mice. (mean ± SD; n=8). *& ^#^ *p*<0.05, ** & ^##^ *p*<0.01, *** *p*<0.001. The asterisk and ^#^ symbols indicate a comparison to the control and CIS-intoxicated groups, respectively.

The level of TNF-α was increased in the CIS-intoxicated group and the CIS + NG group compared to the control group (*p*=0.0001 and *p*=0.0048, respectively). Additionally, the level of TNF-α in the CIS + NG group was significantly decreased compared to the CIS-intoxicated group (*p*=0.0001). NF-κB level was increased in the CIS-intoxicated group compared to the control group (*p*=0.0001), and it significantly decreased in the CIS + NG group compared to the CIS-intoxicated group (*p*=0.0001) (Figure 3).

### Protein Levels of p38 MAPK

**Figure 4:**
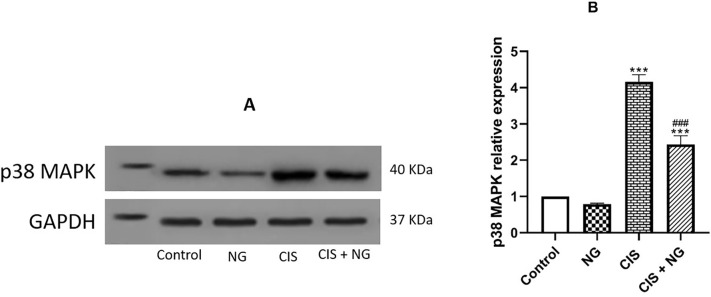
Representative western blot analysis of p38 MAPK expression of testicular tissues, showing protein bands of each group (A) and graphs (B). (mean ± SD; n=8). *& ^#^ p<0.05, ** & ^##^ *p*<0.01, *** *p*<0.001. The asterisk and ^#^ symbols indicate a comparison to the control and CIS-intoxicated groups, respectively.

The concentration of p38 MAPK was significantly increased in both CIS-intoxicated and CIS + NG groups compared to the control group (*p*=0.0001, both). Also, the concentration of p38 MAPK in the CIS + NG group was significantly lower than that in the CIS-intoxicated group (*p*=0.0001) (Figure 4).

### Bax and Bcl-2 Expression

**Figure 5:**
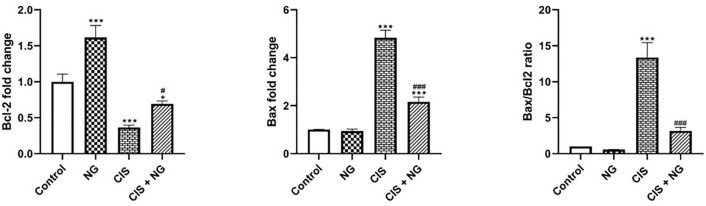
Effect of naringenin on testicular expression of Bax and Bcl-2 in CIS-treated mice. (mean ± SD; n=8). *& ^#^ *p*<0.05, ** & ^##^ *p*<0.01, *** *p*<0.001. The asterisk and ^#^ symbols indicate a comparison to the control and CIS-intoxicated groups, respectively.

Bax was significantly overexpressed in the CIS-intoxicated and CIS + NG groups compared to the control group (*p*=0.0001 and *p*=0.0003, respectively). Additionally, Bax expression decreased significantly in the CIS + NG group compared to the CIS-intoxicated group (*p*=0.0001). The expression of Bcl-2 increased significantly in the NG group compared to the control group (*p*=0.0004). Bcl-2 decreased in the CIS-intoxicated and CIS + NG groups compared to the control (*p*=0.0003 and *p*=0.0264, respectively). furthermore, Bcl-2 was significantly upregulated in the CIS + NG group compared to the CIS-intoxicated group (*p*=0.0201) (Figure 5).

### Histopathological Assessments

**Figure 6:**
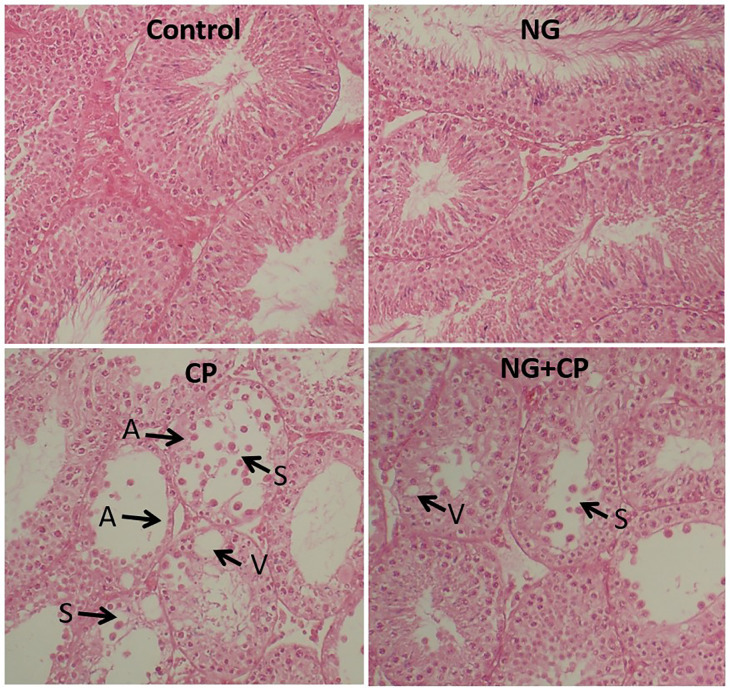
Light microscopy of testicular tissue from the control and experimental groups (V: vacuole, A: atrophy, S: shredded epithelium); H&E staining; Magnifications: ×250.

As shown in Figure 6, the CP-intoxicated group exhibits vacuolization of the germinal epithelium, aligned with the atrophy of the seminiferous tubules. The NG and control groups displayed nearly identical heights of seminiferous epithelium (SEH) and diameters of seminiferous tubules (STD). Both the STD and SEH significantly declined in the CIS-intoxicated group compared to the control group (*p*<0.0001). However, these parameters improved in the CIS + NG group (*p*<0.0001).

**Figure 7:**
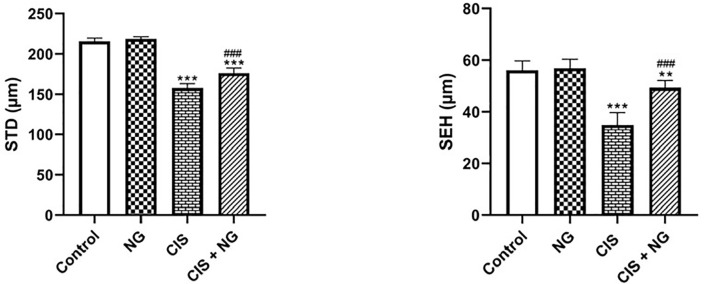
Seminiferous tubule diameter (STD) and seminiferous epithelium height (SEH) in different groups. Values expressed as mean ± SD for six mice. (mean ± SD; n=8). *& ^#^ *p*<0.05, ** & ^##^ *p*<0.01, *** *p*<0.001. The asterisk and ^#^ symbols indicate a comparison to the control and CIS-intoxicated groups, respectively.

**Figure 8:**
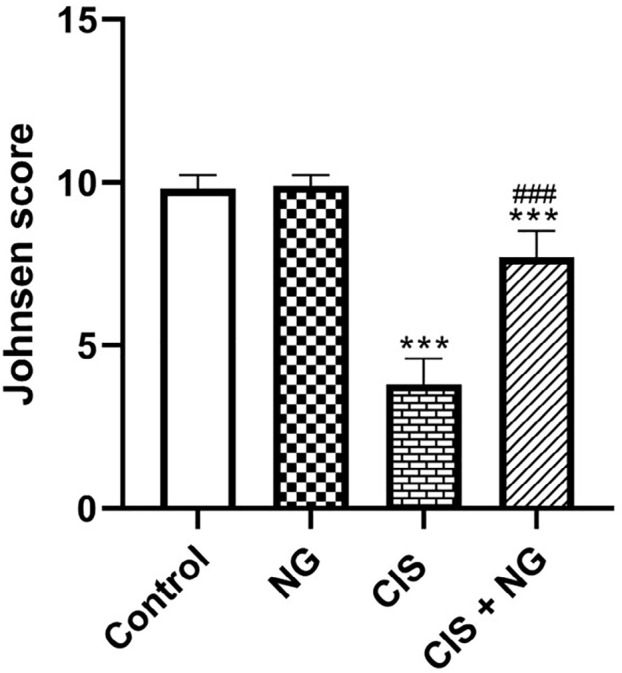
Johnsen scored assessments in the different groups (mean ± SD; n=8). *& ^#^ *p*<0.05, ** & ^##^ *p*<0.01, *** *p*<0.001. The asterisk and ^#^ symbols indicate a comparison to the control and CIS-intoxicated groups, respectively.

Notable changes were detected in the mean Johnsen score. The score significantly decreased in the CIS group compared to the control group (*p*<0.0001). In contrast, the score significantly improved in the CIS + NG group compared to the CIS-intoxicated group (*p*<0.0001) (Figures 7 and 8).

## DISCUSSION

CIS is a widely used chemotherapy drug for treating a variety of cancer types. However, its administration can result in cytotoxicity, which may limit its clinical application (Chtourou *et al*., 2016). In the current study, we evaluated the protective effects of NG against inflammatory, oxidative, and apoptotic damage induced by CIS in testicular tissue (A A Aly & G Eid, 2020).

CIS-induced oxidative stress causes an imbalance between the generation of ROS and the levels of antioxidant enzymes (Ekinci Akdemir *et al*., 2019). The increased production of ROS causes the testis to become more sensitive to the toxic effects of CIS. This oxidative stress leads to testicular damage, apoptosis, DNA damage, and male reproductive dysfunction, ultimately resulting in infertility (Altındağ & Meydan, 2021). The findings of this study revealed a notable rise in oxidative stress levels and a reduction in TAC, consistent with numerous prior research studies (Afsar *et al*., 2017; Eren *et al*., 2020; Ramkumar *et al*., 2021). The NG treatment reduces the free radicals generated by CIS and maintains TAC activities close to those of the control group (Chtourou *et al*., 2016). This effect may be attributed to NG’s ability to suppress lipid peroxidation, decrease oxidative stress by binding to free radicals, and increase the production of antioxidants (Adana *et al*., 2018; Fouad *et al*., 2019).

Enhancing ROS production triggers cell apoptosis (Gach *et al*., 2015). The balance between Bcl-2 and Bax proteins determines whether apoptosis is inhibited or permitted. Bcl-2 is an anti-apoptotic factor that helps maintain the integrity of the mitochondrial membrane (Yang *et al*., 2015). However, Bax is a pro-apoptotic factor that increases in response to oxidative stress, disrupting membrane permeability (Ding *et al*., 2015). The cytotoxicity of CIS is regulated by activating mitochondrial apoptosis and increasing the Bax/Bcl-2 ratio (Türk *et al*., 2011). Consistent with previous studies, this research found that the level of apoptosis in the CIS-intoxicated group increased significantly, and this process was improved by the administration of (Chtourou *et al*., 2016; Ekinci Akdemir *et al*., 2019; Gelen & Şengül, 2020; Rahimi *et al*., 2022).

CIS triggers several cascades by upregulating cytokines such as TNF-α, IL-1β, and IL-6. TNF-α, ultimately resulting in the activation of the NF-κB pathway. NF-κB plays a crucial role in regulating the inflammatory response by promoting the transcription of various pro-inflammatory mediators and regulating the biology of neutrophils, macrophages, and lymphocytes (Humanes *et al*., 2017; Pabla & Dong, 2008; Ramesh & Reeves, 2002). NF-κB and the associated inflammatory regulators are vital for spermatogenesis, testicular steroidogenesis, and semen production (Kucukler *et al*., 2020). The results of this study revealed that CIS causes tissue damage by elevating the levels of TNF-α and subsequently activates NF-κB in testicular tissue. These findings are consistent with previous studies (Hassanein *et al*., 2021; Sioud *et al*., 2020). We also evaluated the NG’s ability to inhibit the upregulation of pro-inflammatory cytokines. Our findings demonstrated that NG can effectively inhibit the production of cytokines, like TNF-α, IL-1β, IL-6, and inactivate the NF-κB signaling pathway, which is in line with earlier research (Pinho-Ribeiro *et al*., 2016; Yang *et al*., 2021).

Oxidative stress and the upregulation of ROS can trigger the activation of p38 MAPKs, initiating inflammatory and apoptotic pathways by increasing the Bax/Bcl-2 ratio (Fouad *et al*., 2020; Lim *et al*., 2019; Liu *et al*., 2018). MAPK signaling pathways have been implicated in NF-κB-dependent inflammatory mediators (Ma *et al*., 2015). It has been confirmed that CIS activate MAPK family, which includes three significant cascades of serine/threonine kinase proteins: p38, JNK and ERK, which play an essential role in regulating cell proliferation, differentiation, and the inflammatory response (Malik *et al*., 2015; Wang *et al*., 2020). In addition, CIS activates the p38 and induces cells to produce ROS, IL-1β, and TNF-α, leading to apoptosis and inflammation (Hsu & Wen, 2002). CIS enhances p38 activation, promoting the translocation of NF-κB to the nucleus and stimulating TNF-α production (Yamakawa *et al*., 1999). Prior research has shown that neutralizing pro-inflammatory cytokines associated with MAPKs can effectively reduce the cytotoxic effects of CIS. NG has been known to modulate p38 MAPK signaling pathways, inhibiting oxidative stress, inflammation, and apoptosis (Fouad *et al*., 2020; Gelen *et al*., 2022).

Histopathological assessments in the CIS-intoxicated group revealed a decrease in the thickness of the germinal epithelium, dissociation of spermatogenic cell layers, interstitial edema, and the presence of vacuoles, along with giant spermatid cells within the lumen of degenerated seminiferous tubules. These findings align with previous studies (Abdel-Latif *et al*., 2022; Hassanein *et al*., 2021; Khamis *et al*., 2023). The histological changes in the germinal epithelium are attributed to the loss of maturation in germinal cells and the interruption of spermatogenesis during the early stages, which is due to the inhibition of B-spermatogonia mitosis, indicating an extension of the G1 phase of the cell cycle (Ijaz *et al*., 2020). Furthermore, germinal epithelial atrophy is related to abnormalities in Leydig cells and reduced testosterone concentrations (Prihatno *et al*., 2018; Reddy *et al*., 2016). It has been shown that serum levels of testosterone can be significantly reduced by downregulating the hypothalamic-pituitary-gonadal axis, which affects steroidogenesis and testosterone synthesis (Fouad *et al*., 2019; Khamis *et al*., 2020). However, bioflavonoid treatment improves testosterone production by upregulating steroidogenic genes and enhancing antioxidant capabilities (Can *et al*., 2022; Khamis *et al*., 2023). Moreover, NG maintains normal levels of spermatogenesis, aligning with previous research findings (Elsawy *et al*., 2022; Fouad *et al*., 2019).

## CONCLUSION

In summary, NG has demonstrated significant protective effects against CIS-induced oxidative stress, apoptosis, and inflammation in testicular tissue integrity. This suggests its potential as a therapeutic agent for preserving male fertility during chemotherapy by restoring antioxidant levels, reducing the oxidative stress index, normalizing inflammatory and apoptotic parameters, and improving morphometric parameters.
